# Ending AIDS by 2030: the importance of an interlinked approach and meaningful youth leadership

**DOI:** 10.1002/jia2.25061

**Published:** 2018-02-27

**Authors:** Hayley S Gleeson, Carlo André Oliveras Rodriguez, Luann Hatane, Doortje‘t Hart

**Affiliations:** ^1^ International Planned Parenthood Federation London United Kingdom; ^2^ Adolescent HIV Treatment Coalition Geneva Switzerland; ^3^ Paediatric‐Adolescent Treatment Africa (PATA) Cape Town South Africa; ^4^ Aidsfonds Amsterdam the Netherlands

**Keywords:** youth leadership, SDGs, interlinkages, participation, HIV, adolescents

## Abstract

**Introduction:**

This commentary by authors from the Adolescent HIV Treatment Coalition calls for action to improve advocacy and service delivery for young people by leveraging the interlinkages between HIV and the broader development agenda. The 2030 Agenda for Sustainable Development includes target 3.3 on ending the AIDS epidemic by 2030, and along with the 2016 Political Declaration on HIV and AIDS, this has led to a global renewal of political commitment to the HIV response. However, young people are still being left behind, and to provide an equitable and sustainable response to HIV we must ensure that we are meeting the needs of the 3.9 million young people living with HIV, and the millions more at risk.

**Discussion:**

While HIV has its own target within the 2030 Agenda, efforts to end AIDS are inextricable from other goals and targets, such as on poverty eradication, education, gender equality and peace. To tackle HIV we must work beyond target 3.3 and provide a comprehensive response that addresses the underlying structural inequalities that impact adolescents and young people, ensuring that we enable the meaningful engagement of youth and adolescents as partners and leaders of sustainable development and the HIV response. Finally, it is necessary to collect better disaggregated data and evidence on the HIV epidemic among adolescents, as well as on best practices for supporting them.

**Conclusions:**

Ending the AIDS epidemic among adolescents and young people (aged 10 to 24) by 2030 is possible. However, it requires an integrated, multi‐sectoral response to HIV which pays attention to the social determinants that put adolescents at risk and fuel the epidemic. Positioning efforts to end AIDS among young people within the broader 2030 Agenda and building youth leadership will contribute to building a more healthy, equitable and sustainable society for all.

## 1 Introduction

In 2015, UN Member States adopted the 2030 Agenda for Sustainable Development, a complex and ambitious human rights‐based framework with 17 Sustainable Development Goals (SDGs) and 169 corresponding targets which will drive global priorities until 2030 1. Target 3.3 calls for the end of the AIDS epidemic, and builds on the significant progress made under the Millennium Development Goals, which reduced new HIV infections by 40 per cent from 2000 to 2013 2. However, inequalities remain, and women, young people and key populations are disproportionately affected. In 2015, there were 250,000 new HIV infections among adolescents, two thirds of which were among adolescent girls 3. Treatment coverage has been one of the HIV response's biggest successes: while just 375,000 people living with HIV were on antiretroviral therapy (ART) in 2003 2, this had reached 19.5 million by 2016 4. However, progress for young people aged 15 to 24 still lags behind, and this group is less likely to be diagnosed or on treatment than adults 5.

There is growing evidence that medical interventions alone are not sufficient to tackle HIV 6. Efforts to end AIDS must address the structural barriers which restrict access to treatment and care for the 3.9 million young people aged 15 to 24 living with HIV 4, as well to prevention services for the millions more at risk. This especially includes harmful gender norms which systematically disadvantage women and restrict them from exercising their rights. In this commentary, authors from the Adolescent HIV Treatment Coalition argue that progress in the HIV response for young people can be improved by leveraging the links between HIV and other SDG targets on poverty eradication, education, health, gender equality, and peace and justice. Most importantly, we affirm the right of young people to actively participate in decisions that affect their lives, and emphasize the importance of involving young people, especially those living with and most affected by HIV, at all levels of the HIV response and in SDG implementation. Finally, we call for better data and evidence on how the HIV epidemic impacts young people, and on the best practices to support them, to ensure effective and sustainable programming.

## 2 Discussion

Young people do not live single‐issue lives, and their access to HIV information and services is intimately connected to social, political and economic factors. The SDGs are “integrated and indivisible” from each other 1, and in our programming we must strive to see HIV and target 3.3 within a broader lens of social, economic and environmental development. Many SDG targets can support progress towards the HIV response and the 2030 Agenda, and interlinked advocacy and programming has the potential to benefit both simultaneously (Table 1).

**Table 1:** Select SDG targets that connect to the HIV response 1

Goal	Selected target(s)
1: End poverty in all its forms everywhere	1.3: Implement nationally appropriate social protection systems and measures for all
3: Ensure healthy lives and promote wellbeing for all at all ages	3.3: End the epidemics of AIDS, tuberculosis, malaria and neglected tropical diseases and combat hepatitis, water‐borne diseases and other communicable diseases 3.7: Ensure universal access to sexual and reproductive healthcare services, including for family planning, information and education
4: Ensure inclusive and equitable quality education and promote lifelong learning opportunities for all	4.1: Ensure that all girls and boys complete free, equitable and quality primary and secondary education 4.7: Ensure that all learners acquire the knowledge and skills needed to promote sustainable development, including, among others, through education for sustainable development and sustainable lifestyles, human rights, gender equality, promotion of a culture of peace and non‐violence
5: Achieve gender equality and empower all women and girls	5.1: End all forms of discrimination against all women and girls everywhere 5.3: Eliminate all harmful practices, such as child, early and forced marriage and female genital mutilation 5.6: Ensure universal access to sexual and reproductive health and reproductive rights
10: Reduce inequality within and among countries	10.2: Empower and promote the social, economic and political inclusion of all, irrespective of age, sex, disability, race, ethnicity, origin, religion or economic or other status 10.3: Ensure equal opportunity and reduce inequalities of outcome, including by eliminating discriminatory laws, policies and practices and promoting appropriate legislation, policies and action in this regard
16: Promote peaceful and inclusive societies for sustainable development, provide access to justice for all and build effective, accountable and inclusive institutions at all levels	16.6: Develop effective, accountable and transparent institutions at all levels 16.7: Ensure responsive, inclusive, participatory and representative decision making at all levels
17: Strengthen the means of implementation and revitalize the Global Partnership for Sustainable Development	17.18: Enhance capacity‐building support to developing countries to increase significantly the availability of high‐quality, timely and reliable data disaggregated by income, gender, age, race, ethnicity, migratory status, disability, geographic location and other characteristics relevant in national contexts

N.B. targets have been paraphrased. SDG, Sustainable Development Goals.

Eradicating poverty is highlighted in the preamble of the 2030 Agenda as “an indispensable requirement for sustainable development,” and is a prerequisite to improving health 1. Poverty and HIV have a complex and bi‐directional relationship; both are influenced by the same systemic inequalities and power dynamics. Healthcare services, schools and sanitation are often inaccessible for people living in poverty, who may be living in remote or conflict areas 7. Poverty disproportionately affects women, who are less likely than men to participate in the labour market, and shoulder the majority of unpaid care work 8. Poverty can also be a driver of transactional sex – the exchanging of sex for material goods including food, gifts and cash – which in turn increases the risk of HIV acquisition 9.

Social protection systems, which attempt to reduce social and economic vulnerability of the most marginalized groups, further progress towards target 1.3, and can reduce HIV risk behaviours 10, as well as improve adherence to ART for adolescents 11. While cash transfers alone may have some impact on HIV risk, combining cash with “care” interventions – psychosocial support, such as from parents or teachers – more effectively reduce HIV risk for male and female adolescents 12, 13, 14, 15. Social protection systems have been shown to improve adolescent health outcomes across 12 SDG indicators, spanning the goals on hunger, health, education, gender equality and peaceful societies 10. There is clear potential for organizations working on poverty reduction to use social protection interventions to support the achievement of target 3.3 for adolescents by reducing HIV risk behaviours and increasing treatment adherence.

Links between HIV and sexual and reproductive health and rights (SRHR), covered by targets 3.7 and 5.6, are widely acknowledged. Poor sexual health has many of the same structural drivers as HIV, including gender‐based violence, inequality and criminalization of behaviours including same‐sex relationships, sex work and drug use 16. Bi‐directional integration of HIV and SRHR programmes can lead to increased uptake of services, increased condom use, better HIV testing outcomes and reduction in HIV‐related stigma, as well increasing cost‐effectiveness 17. Integration of HIV services into maternal health and family planning services may also be a useful entry point for women 18, 19. For young people, integrating HIV and SRHR services has been shown to improve health outcomes due to increased uptake of services, the ability to access multiple services at one time, and improved healthcare provider attitudes 20. Targets 3.3 and 3.7 should be tackled in tandem with one another, understanding that young people's access to SRH and family planning services can be improved by combining these services with HIV prevention, treatment and support.

Strengthening education systems under SDG 4 can have significant impacts on HIV. Pettifor et al. showed that young women who had missed a school grade or who had missed more than 4 days of school in the last month were significantly more likely to be living with HIV than those who had not, indicating that remaining in school can protect young girls against HIV infection 21. There is also evidence that this effect goes beyond the individual, with a study from Zambia showing that increased educational attainment at neighbourhood level was linked to lower HIV prevalence among young women 22. Formal education is a key pathway to economic empowerment for young women and girls, contributing to higher self‐esteem, increased uptake of SRHR services, and delayed marriage and childbirth 23, 24. However, there are immense economic and social barriers that affect school attendance, especially for young girls who may be forced into early marriage or domestic work. To end the HIV epidemic among adolescents and youth, investments in education, and in addressing the structural barriers that keep young girls out of school, are therefore critical.

Despite the fact that AIDS is a leading cause of death for young people globally, education on HIV is dangerously inadequate. Only 36% of young men and 30% of young women aged 15 to 24 have comprehensive knowledge of HIV and how to prevent it 5. Comprehensive sexuality education (CSE) is a crucial intervention for the HIV response, which can support progress towards targets 3.3, 3.7 and 4.7. CSE can contribute to reducing sexually transmitted infections (including HIV) and unintended pregnancy, increasing condom use, increasing self‐esteem, and promoting gender equality and more equitable social norms, which in turn can improve health outcomes 25. We must expand access to CSE, in particular for young women and girls, key populations and out‐of‐school youth, as this is a key pathway to prevent HIV, reduce HIV stigma and link young people to care and support. Engaging young people in the design, implementation and evaluation of CSE programmes can ensure that material is taught in age‐appropriate, culturally sensitive ways, which adequately meet the needs of young people.

It will be impossible to end AIDS without addressing Goal 5 on gender inequality, a primary structural driver of the epidemic. Patriarchal gender norms, cultural beliefs and unequal power dynamics leave women with limited economic opportunities, dependent on male partners, and frequently subjected to intimidation and violence 24. Women are often unable to make decisions related to their SRHR, and in some cases discriminatory spousal consent laws prevent women from independently accessing SRH or HIV care 26. Almost a third of women worldwide have experienced physical or sexual violence by a partner, and women who have been abused by their partners are more likely to acquire HIV compared to women who have not experienced abuse 27.

Globally, around 700 million women alive today were married before they were 18, with almost a third of those married before they were 15 28. Girls forced into early marriage have restricted access to education and employment, and usually do not have the power to negotiate safe sex, increasing their risk and vulnerability to HIV and other STIs 28, 29. Transgender women may be more likely to experience intimate partner violence 30 and are almost 49 times more likely to acquire HIV 31, yet are often left out of programming and decision‐making. Gender equality for women and for transgender, intersex and non‐binary people is central to ending the AIDS epidemic, and the importance of tackling these gendered barriers to sexual and reproductive health information and services cannot be overstated. Achieving either Goal 3 or 5 for young people will be impossible without significant progress in the other, and HIV programming must understand how gender inequalities influence the HIV epidemic, and how women and girls seek out HIV prevention, testing and treatment services.

Service delivery, advocacy and programming for young people must take place within an enabling legal and policy environment that recognizes young people's right to live free from violence and discrimination, and to safely exercise their rights, including their reproductive rights. Progressive laws, which protect human rights and ensure access to clinical care and other forms of support, are necessary to end the HIV epidemic. Target 10.3 calls for the elimination of discriminatory laws and policies to reduce inequalities of outcome. In 72 countries, young people under a certain age must seek parental consent before accessing one or more SRHR service 26. Such policies have been widely condemned by the global health community, including the Committee on the Rights of the Child, and act as a significant barrier to young people's access to care. Globally, 44% of new HIV infections occur in key populations – sex workers, people who inject drugs, transgender people and men who have sex with men – and their sexual partners 5. Criminalization of behaviours like sex work and drug use perpetuates stigma and restricts uptake of services. HIV programming should therefore include political advocacy at the highest levels to repeal and reform all laws that restrict these rights, and also address target 16.6 to strengthen justice systems and improve young people's access to formal redress and accountability mechanisms for rights violations.

Target 16.7 calls for inclusive, representative and decision‐making at all levels – and this includes in the design, implementation and monitoring of HIV interventions. As we work towards ending the HIV epidemic for adolescents and young people, it is our responsibility to use a bottom‐up approach, ensuring that advocacy and programming reflects the voices of young people who are most at risk of HIV. Meaningful youth engagement in design and delivery of HIV interventions can lead to increased acceptance from other young people, as well as higher levels of uptake and effectiveness 31. It also fulfils young people's right under the Convention on the Rights of the Child to freely express their views on topics that affect them, and to have those views listened to.

**Figure 1:**
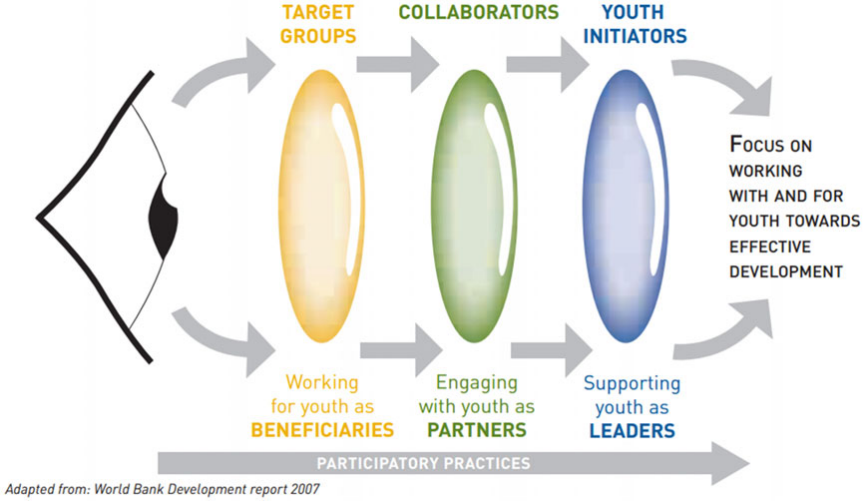
The three‐lens approach to youth participation. This model outlines how young people should be engaged in development programmes as beneficiaries, partners and leaders 32.

In 2010, the Youth Civil Society Working Group of the UK Department for International Development in conjunction with other civil society organizations developed the “three‐lens” approach to youth participation, which articulates three dimensions of working with young people to ensure effective, sustainable development programmes (Figure 1). Good practice participation considers all three lenses and is implemented throughout the lifecycle of development. Working *for* youth as beneficiaries (the first lens) sets the scene for working *with* youth as partners (the second lens), where young people work collaboratively throughout the intervention in a supporting role. Ultimately, we must aim to equip young people with the skills and resources to design and implement programmes which are bottom‐up and youth‐led (the third lens). This includes technical as well as financial support for community‐led and youth‐led service delivery.

Establishing mechanisms for sustainable and meaningful youth engagement in development furthers global progress towards targets 16.7 and target 10.2 on political inclusion, while also supporting the achievement of target 3.3 in a comprehensive and collaborative way. We must pay particular attention to facilitating participation of young people living with, and most affected by, HIV, including adolescent girls and young women, LGBTI people, people with disabilities and migrants, who are further excluded from participating in decision making. Efforts should be made to fit engagement opportunities into the daily lives of young people, for example at schools, clinics and community centres.

The 17th and final SDG focuses on the means of implementation of the 2030 Agenda, and is an overarching goal which draws attention to the need for sustainable financing, strong cross‐sector partnerships and capacity‐building, among other systemic issues. Here we particularly note target 17.18, on “high‐quality, timely and reliable” disaggregated data. We will not end AIDS among young people without an accurate and comprehensive picture of what is going on in young people's lives, the factors that increase their risk and vulnerability to HIV and violence, and the key barriers to progress. There is an urgent need for data on young people – in particular 10 to 14 and 15 to 19 year olds – that is disaggregated by age, sex and key population status. It is also important to support young people to develop skills to collect and analyze their own data, as this can give insight into their lives that is not reflected in large scale surveys, and also allows a transition to the third lens of youth engagement, with young people designing and implementing programmes based on their unique needs and experiences.

## 3 Conclusions

The end of AIDS is within reach, but structural barriers hinder our progress. Reframing the HIV response within a broader context of the 2030 Agenda can support progress towards the end of AIDS as well as towards poverty eradication, education and gender equality.

We are all accountable for achieving the 2030 Agenda and leaving no one behind. As the Adolescent HIV Treatment Coalition, we call on all development stakeholders to continue building the evidence base on interlinkages of HIV across the 2030 Agenda, and how best to provide holistic, cross‐sectoral programming that recognizes the unique challenges that young people face. We must create enabling legal and policy environments that uphold young people's rights, and uproot the systemic inequalities that keep them at risk and inadequately served. We must facilitate inclusive and participatory decision‐making, recognizing that young people can collaborate with governments to achieve the SDGs together, and invest in young people not just as beneficiaries of HIV programmes, but as partners and leaders in the response. Finally, we must ensure a fully‐funded, youth‐ and gender‐sensitive HIV response, and build capacity of young people to hold their governments accountable, equipping them with the skills and tools to carry the HIV response forward in an effective and sustainable way.

## Competing interests

The authors have no competing interests to declare.

## Authors’ contributions

HG contributed to the concept, research, writing and revision. COR contributed to the concept, research, writing and revision, and coordination of authors. LH contributed to the concept, technical input and revision of drafts. DTH contributed to the concept, technical input and revision of drafts. All authors have read and approved the final manuscript.
